# Supplementary material for: Cryo-EM Structure of Human ATAD2B Reveals a Hexameric Organization Contributes to ATPase Activity and Substrate Coordination
Source: bioRxiv. 2026 Apr 3:2026.04.02.716110. Preprint. [Version 1] doi: 10.64898/2026.04.02.716110 (PMC13060104; doi:10.64898/2026.04.02.716110)
Supplement: 1 [file NIHPP2026.04.02.716110v1-supplement-1.pdf]

bioRxiv preprint doi: https://doi.org/10.64898/2026.04.02.716110; this version posted April 3, 2026. The copyright holder for this preprint (which was not certified by peer review) is the author/funder, who has granted bioRxiv a license to display the preprint in perpetuity. It is made available under aCC-BY 4.0 International license.

Supplemental Information

Supplementary Table 1. Cryo-EM data collection, refinement, & validation statistics

Data Collection and Processing	
**EMDB Code**	*available after acceptance*
**PDB Code**	*available after acceptance*
**Electron Microscope**	Titan Krios
**Camera**	Gatan K3
**Magnification**	105000x
**Voltage**	300 kV
**Nominal defocus range (µm)**	-0.8 to -2.5
**Total electron dose (e-/Å^2)**	50.03
**Pixel Size (Å)**	0.835
**Exposure time (sec)**	23.3
**Number of frames per movie**	50
**Automation software**	Latitude S ver. 3.44 (Gatan)
**Micrographs collected**	4,870
**Data processing software**	cryoSPARC v.4.5.3
**Symmetry imposed**	None: Default C1
**Total extracted particles**	1,363,273
**Total refined particles**	213,605
**Final particles**	213,605
**Map resolution (FSC=0.143/Å)**	3.0
**Local resolution range (Å)**	3-6.5
**Refinement**	
**Initial Model (AlphaFold code)**	AF- Q9ULI0-F1-v4
**Refinement package**	Phenix 1.21.2-5419
**Model resolution (FSC=0.5/Å)**	3.2
**Map sharpening B factor (Å^2)**	NA
**Map CC**	0.86
**Model composition**	
Non-hydrogen atoms	26769
Protein residues	3301
Ligands	6
**B factors (Å^2) (min/max/mean)**	
Protein	16.77/260.85/112.36
Ligands	15.72/249.48/97.34
**Root mean square deviations**	
Bond lengths (Å)	0.003

bioRxiv preprint doi: https://doi.org/10.64898/2026.04.02.716110; this version posted April 3, 2026. The copyright holder for this preprint (which was not certified by peer review) is the author/funder, who has granted bioRxiv a license to display the preprint in perpetuity. It is made available under aCC-BY 4.0 International license.

Bond angles (°)	0.459
**Validation**	
**MolProbity score**	1.35
Clash score	2.51
Poor rotamers (%)	0
**C-beta deviations**	NA
**CaBLAM outliers**	3.74
**Ramachandran plot (%)**	
Favored	95.48
Allowed	4.52
Outlier	0

bioRxiv preprint doi: https://doi.org/10.64898/2026.04.02.716110; this version posted April 3, 2026. The copyright holder for this preprint (which was not certified by peer review) is the author/funder, who has granted bioRxiv a license to display the preprint in perpetuity. It is made available under aCC-BY 4.0 International license.


```
ATAD2B   MVNTRKSSLRLLGSKSPGPGPGPGAGAEPGATGGSSHFISSRTRS-----SKTRAASCPA   55
ATAD2    MVVL-RSSLELHNH---------SAASATGSLDLSSDFLSLEHIGRRRLRSAGAAQKKPA   50
Abo1     ------------------------------------------------------------   0
Yta7     --------------------------------------------MARNLRNRRGSDV--E   14

ATAD2B   AKAGGSGGAGVTLDEARKVEVDGSLSDSHVSPPAKRTLKQPDSVCKDKSKS---------   106
ATAD2    ATT-AKAGDGSSVKEVETYHRTRA----------LRSLRKDAQNSSDSSFE--------K   91
Abo1     ----------------------------------------------------------MK   2
Yta7     DASNAKVGYETQIKDEN-----G------IIHTTTRSLRKINYAEIEKVFDFLEDDQVMD   63

ATAD2B   -------RSTGQREEWNLSTGQARL---------TSQPGATLPNGHSGLSLRSHPLRGEK   150
ATAD2    NVEITEQLANGRHFTRQLARQQADKKK------EEHREDKVIPV------TRSLRARNIV   139
Abo1     E-------------EASEHGGSADETQ-ELSPVSDS--SDEMPNNA-KRRRRSQSMIANK   45
Yta7     KDETPVDVTSDEHHNNNQKGDDEDDDVDLVSPHENARTNEELTNER-NLRKRK--AHDPE   120

ATAD2B   KGDGDLSCINGDMEVRKSCRSRKNRFESVNQSLLFDQLVNSTAEAVLQEMDNINIRRNRR   210
ATAD2    QSTEHLHEDNGDVEVRRSCRIR-SRYSGVNQSMLFDKLITNTAEAVLQKMDDMKKMRRQR   198
Abo1     RIHQAFQEDEGDEDWEE-------------------------------EEHKPKA-KRR-   72
Yta7     EDDESFHEEDVDDDEEEEEA---DEF----------------EDEYLDEDSKDNN-RRRR   160

ATAD2B   SGE--V-----ERLRMWTDTEFENMDMY------SRVKRRRKSLRRN-----SY------   246
ATAD2    MRE--L-----EDLGVFNETEESNLNMY------TRGKQKDIQRTDEETTDNQEGS-VES   244
Abo1     -----YNT---RSNESFSEGDDEPFEVSESSALEDELSDSEDSFIRSVRSKPKYKPGTRR   124
Yta7     AADRKFVVPDPDDDEEYDEDDEEGDRISHSA-SSKRLKRANSRRTRS-SRHPETPPPVRR   218

                          ATAD2 Acidic N-terminus
ATAD2B   ---GIQNHHE-VSTEGEEEE---------SQEEDGDIEVEEAEGEENDRPYNLRQRKTVD   293
ATAD2    SEEGEDQEHE-DDGEDEDDEDDDDDDDDDDDDDDEDDEDEEDGEEENQKRYYLRQRKATV   303
Abo1     ST-RLRNRRSQD-----------------------------EEESEEEHRPILRERTSRI   154
Yta7     AL-RSRTRHSRTSNEENDDENDN-SRNEALTLADEIREL--QEDSPIREKRFLRERTKPV   274

ATAD2B   RYQAPPIVPAHQKKR-----ENTLFDIHRSPAR---------------------------   321
ATAD2    YYQAPLEKPRHQRKP-----N-IFYSGPASPARPRY-RLS-------------SAGPRSP   343
Abo1     NYSVPLAFPPVDEMD--GD---PSSQVNQSRSRKTHSEL---------------------   188
Yta7     NYKLPPPLTASNAEEFIDKNNNALSFHNPSPARRGRGGWNASQNSGPTRRLFPTGGPFGG   334

ATAD2B   -------RSHIRRKKHAIHSSDTT----SSDEERFERRKSKSMARARNRCLPMNFRAEDL   370
ATAD2    ------YCKRMNRRRHAIHSSDSTSSSSSEDEQHFERRRKRSRNRAINRCLPLNFRKDEL   397
Abo1     -AITKLLRQQVSSFMPYIDSSG---SESESDNTRI-KK------SSAKTIKALTDPANSG   237
Yta7     NDVTTIFGKN-TNFYNQVPSAF-----SDNNNNKLILDSD----SSDDEILPLGVTPKTK   384
```

bioRxiv preprint doi: https://doi.org/10.64898/2026.04.02.716110; this version posted April 3, 2026. The copyright holder for this preprint (which was not certified by peer review) is the author/funder, who has granted bioRxiv a license to display the preprint in perpetuity. It is made available under aCC-BY 4.0 International license.

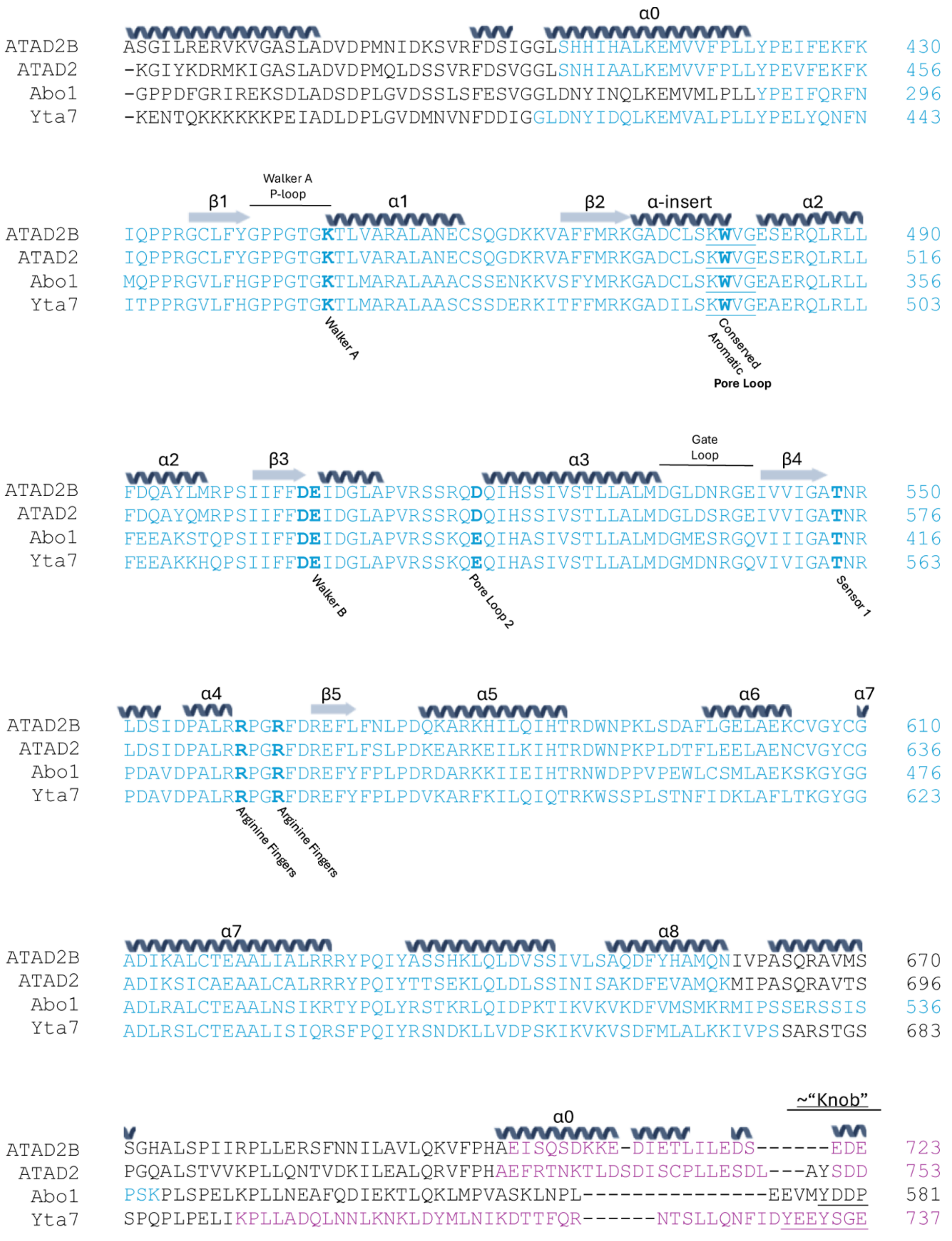

bioRxiv preprint doi: https://doi.org/10.64898/2026.04.02.716110; this version posted April 3, 2026. The copyright holder for this preprint (which was not certified by peer review) is the author/funder, who has granted bioRxiv a license to display the preprint in perpetuity. It is made available under aCC-BY 4.0 International license.

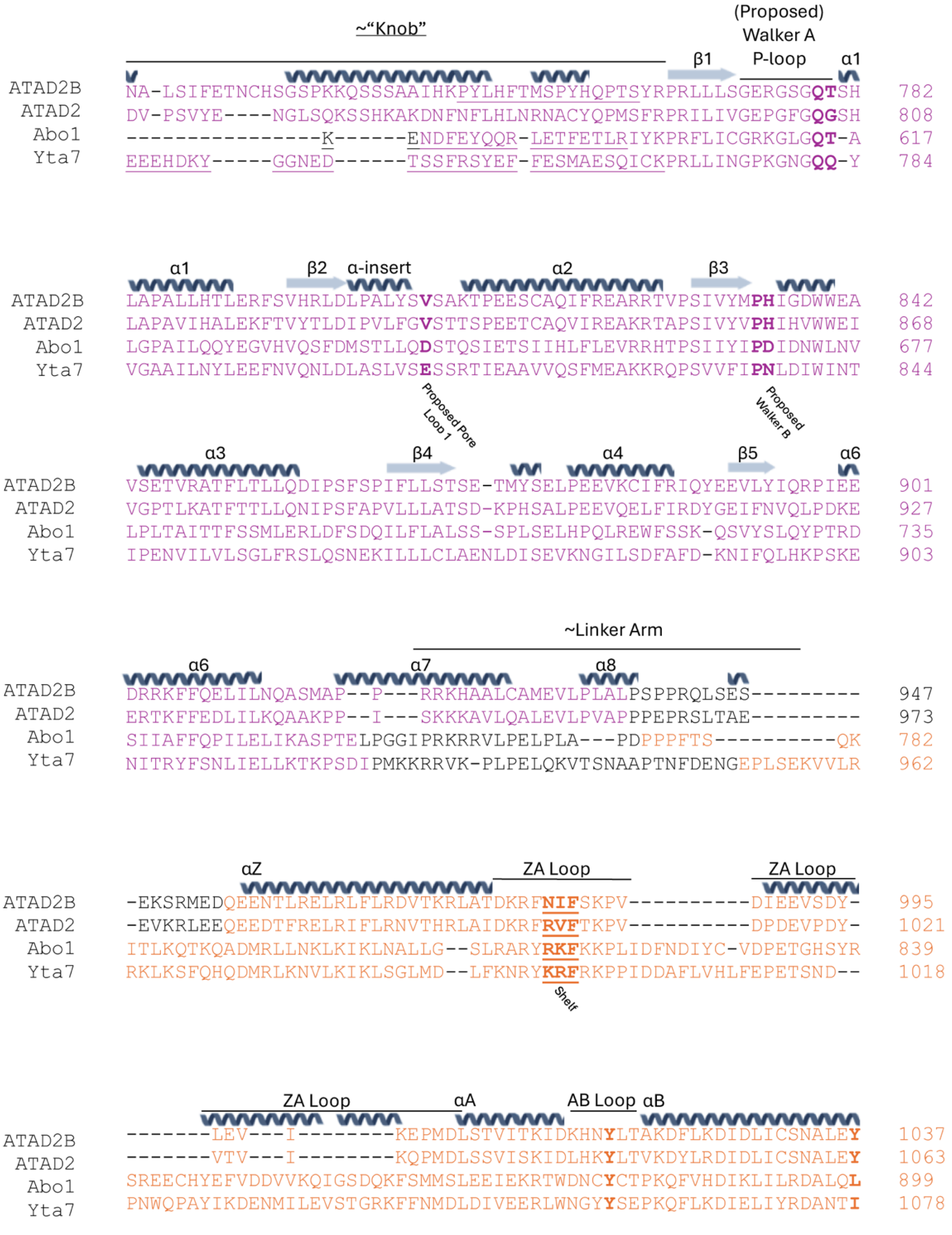

bioRxiv preprint doi: https://doi.org/10.64898/2026.04.02.716110; this version posted April 3, 2026. The copyright holder for this preprint (which was not certified by peer review) is the author/funder, who has granted bioRxiv a license to display the preprint in perpetuity. It is made available under aCC-BY 4.0 International license.

Conserved Tyrosine

Conserved Binding Pocket (Tyrosine)

BC Loop αC αC

```
ATAD2B  NPDKDPGDKIIRHRACTLKDTAHAIIAAELDPEFNKLCEEIKEARIKRGLSVT-SEQIN-   1095
ATAD2   NPDRDPGDRLIRHRACALRDTAYAIIKEELDEDFEQLCEEIQESRKKRGCSSS-KYAPSY   1122
Abo1    E------DSETIKRAQEMYANVLLGVEDMEDDQFSQRCERMALREAERRKLRHGKLQKH-   952
Yta7    G------DRERVIKASEMFANAQMGIEEISTPDFIQECKATRQRDLERQELFLEDE----   1128
```

Conserved Binding Pocket (Asparagine)

Gatekeeper

```
ATAD2B  ----PH----STGARKTETRVEEAFRHKQRNPMDVWHNSANKCAFRVRRKSRRRSQWGKG   1147
ATAD2   YHVMPKQNSTLVGDKRSDPEQNE----KLKTPST---PVACSTPAQLKRKIRKKSNWYLG   1175
Abo1    -----------LDETKADMQ----FTSE--KPSV------DESITEVD--D------AI-   980
Yta7    -------------EKRAAME----LEAK--EQSQ------ENILQEPDLKDNKANEFGV-   1162

ATAD2B  IIKKRKVNNLKKDEEDTKFAD-YENHTEDRKLLENGEFEVSTDCHEENGEETGDLSMTND   1206
ATAD2   TIKKRRKISQAKDDSQNAIDHKIESDTEET-----QD--TSV-DHNETG-NTGESSVEEN   1226
Abo1    -KDGP------------PVLAE---TLT-NSLME-DVGPENVDMDIED------NEIFTN   1016
Yta7    -AAGN------------QLQAQLQTTINTASIVNNSEVPQPIDT-----------NLYKK   1198

ATAD2B  ESSCDIMDL--DQGQRLNNGAGTKENFASTEEESSNESLLVNSSSSLNPEQTSRKETFLK   1264
ATAD2   EK--------------QQNASES-------------KLELRNNSN---------------   1244
Abo1    QSTMSVPSMLVEN-----EESPKPDEYID--------------------QKDKVQSPLLN   1051
Yta7    EIPAAIPSAVDKEKAVIPEDSGANEEYTT---------------------------ELIQ   1231

ATAD2B  GNCLNGEASTDSFEGIPVLECQNGKLEVVSFCDSGDKCSSEQKILLEDQSKEKPETSTEN   1324
ATAD2   -TCNIENELEDS---RKTTACTEL--RDKIACN-GDA-SSSQIIHI----------SDEN   1286
Abo1    GKSPVGVPSEAALRVST-------DVSTNI--------------SSNGRADIPVD--TLI   1088
Yta7    ATCTSEITTDDDERARKEPKENEDSLQTQV--------------TEENFSKIDANTNNIN   1277

ATAD2B  HGDDLEKLEALECSNNEKLEPGSDVEVKDAELDKEGASKVKKYRKLILEQAKTTSL---E   1381
ATAD2   EGKEMCVL------------------------------RMTRARRSQVEQQQLITV---E   1313
Abo1    TSPADVPNN----------AP-------------------TDAHNITSADGHIENIEQEV   1119
Yta7    HVKEIQSVN----------KP-------------------NSLHETV-EKRERSPIPKEV   1307
```

bioRxiv preprint doi: https://doi.org/10.64898/2026.04.02.716110; this version posted April 3, 2026. The copyright holder for this preprint (which was not certified by peer review) is the author/funder, who has granted bioRxiv a license to display the preprint in perpetuity. It is made available under aCC-BY 4.0 International license.


```
ATAD2B     LVPEEPSEPVPPLIVDRERLKKLLDLLVDKSNNLAVDQLERLYSLLSQCIYRHRKDYDKS   1441
 ATAD2     KALAILSQPTPSLVVDHERLKNLLKTVVKKSQNYNIFQLENLYAVISQCIYRHRKDHDKT   1373
  Abo1     V--------FPDLVFDEDRLTPLKQLLIDSTTGFTVDQLLHLHSFLYQIIWNTKSEWNRN   1171
  Yta7     VEPEQGKKSDKELILTPEQIKKVSACLIEHCQNFTVSQLEDVHSSVAKIIWKSKSAWDKT   1367

ATAD2B     QLVEEMERTVHMFETFL--       1458
 ATAD2     SLIQKMEQEVENFSCSR--       1390
  Abo1     SVVDECERAVKEFMINALQ       1190
  Yta7     GTVDEIIKFLSE-------       1379
```

Supplementary Figure 1. Annotated sequence alignment for the ATAD2-like protein family. Multiple sequence alignment of members of the ATAD2-like protein family with published structures. ATAD2B (UniProt ID: Q9ULI0), ATAD2 (UniProt ID: Q6PL18), Abo1 (UniProt ID: O14114), Yta7 (UniProt ID: P40340) were aligned using the Clustal Omega Multi Sequence Alignment online tool (https://www.ebi.ac.uk/jdispatcher/msa/clustalo). Secondary structure prediction was performed using PROMALS3D[73]. The secondary structure elements are indicated above the sequence, and conserved residues or structural motifs are labeled. The sequence alignment is colored by domain as indicated in Figure 1a where the acidic patch in the N-terminal region is shown in red, the AAA1 domain is blue, the AAA2 domain is purple, the bromodomain is orange, and the C-terminal region is green.

bioRxiv preprint doi: https://doi.org/10.64898/2026.04.02.716110; this version posted April 3, 2026. The copyright holder for this preprint (which was not certified by peer review) is the author/funder, who has granted bioRxiv a license to display the preprint in perpetuity. It is made available under aCC-BY 4.0 International license.

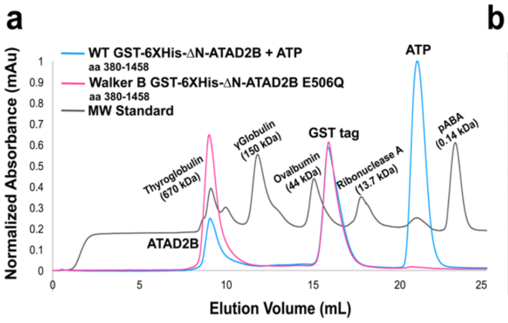

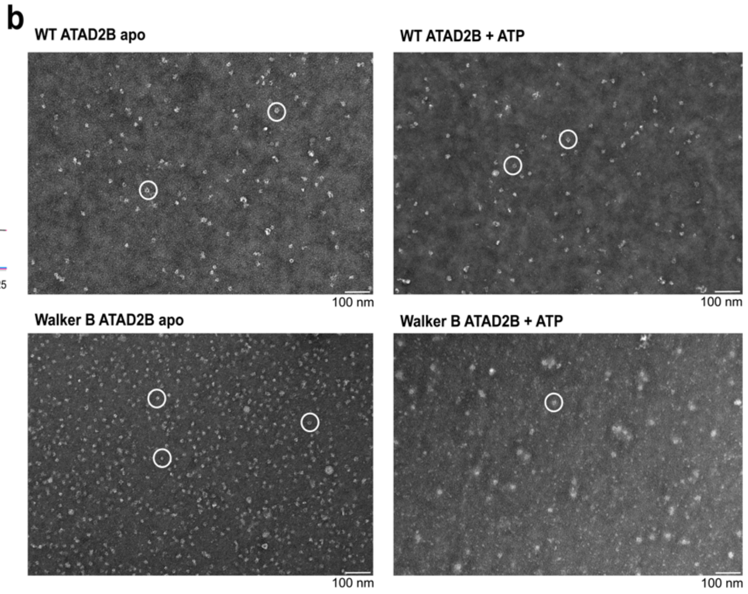

c

Steady-state ATP hydrolysis rates for ATAD2-like proteins	
This study	*ATP/hexamer/sec*
ΔN-ATAD2B WT (aa 380-1458)	0.34 ± 0.01
Walker B ΔN-ATAD2B E506Q (aa 380-1458)	0.19 ± 0.01
	ATP/hexamer/sec
ΔN-ATAD2 WT (aa 403-1390)(Cho et al., 2023)	0.0005
ΔN-Abo1 WT (aa 243-1190)(Cho et al., 2019)	0.83
Yta7 WT (aa 1-1379)(Wang et al., 2023)	0.43

Supplementary Figure 2. Further investigation into ATAD2B oligomerization.
a) Size exclusion chromatography (SEC) traces for the wild-type (WT) GST-His6x-ΔN-ATAD2B (residues 380-1458) (blue) and the Walker B (E506Q) GST-His6x-ΔN-ATAD2B Walker B (residues 380-1458) (pink) proteins are overlayed with the molecular weight (MW) standard (grey). The GST-His6x-ΔN-ATAD2B (residues 380-1458) is referred to as the wild-type ATAD2B in this study for clarity. The expected molecular weight for monomeric ATAD2B is ~150 kDa. **b)** Negative stain electron micrographs captured at 30,000 x on a JEOL 1400 transmission electron microscope (TEM). Hexameric particles are highlighted with a white circle. **c)** Table comparing the steady-state ATP hydrolysis rates of WT ATAD2B and Walker B ATAD2B from this study to the published ATP hydrolysis rates for Abo1[22], Yta7[25], and ATAD2[26]. The EnzChek^{TM} phosphate assay kit used in our study was also utilized to measure the rates of ATAD2[26] and Abo1[22]. The Yta7 results were determined by measuring the phosphate released from ATP hydrolysis[25].

bioRxiv preprint doi: https://doi.org/10.64898/2026.04.02.716110; this version posted April 3, 2026. The copyright holder for this preprint (which was not certified by peer review) is the author/funder, who has granted bioRxiv a license to display the preprint in perpetuity. It is made available under aCC-BY 4.0 International license.

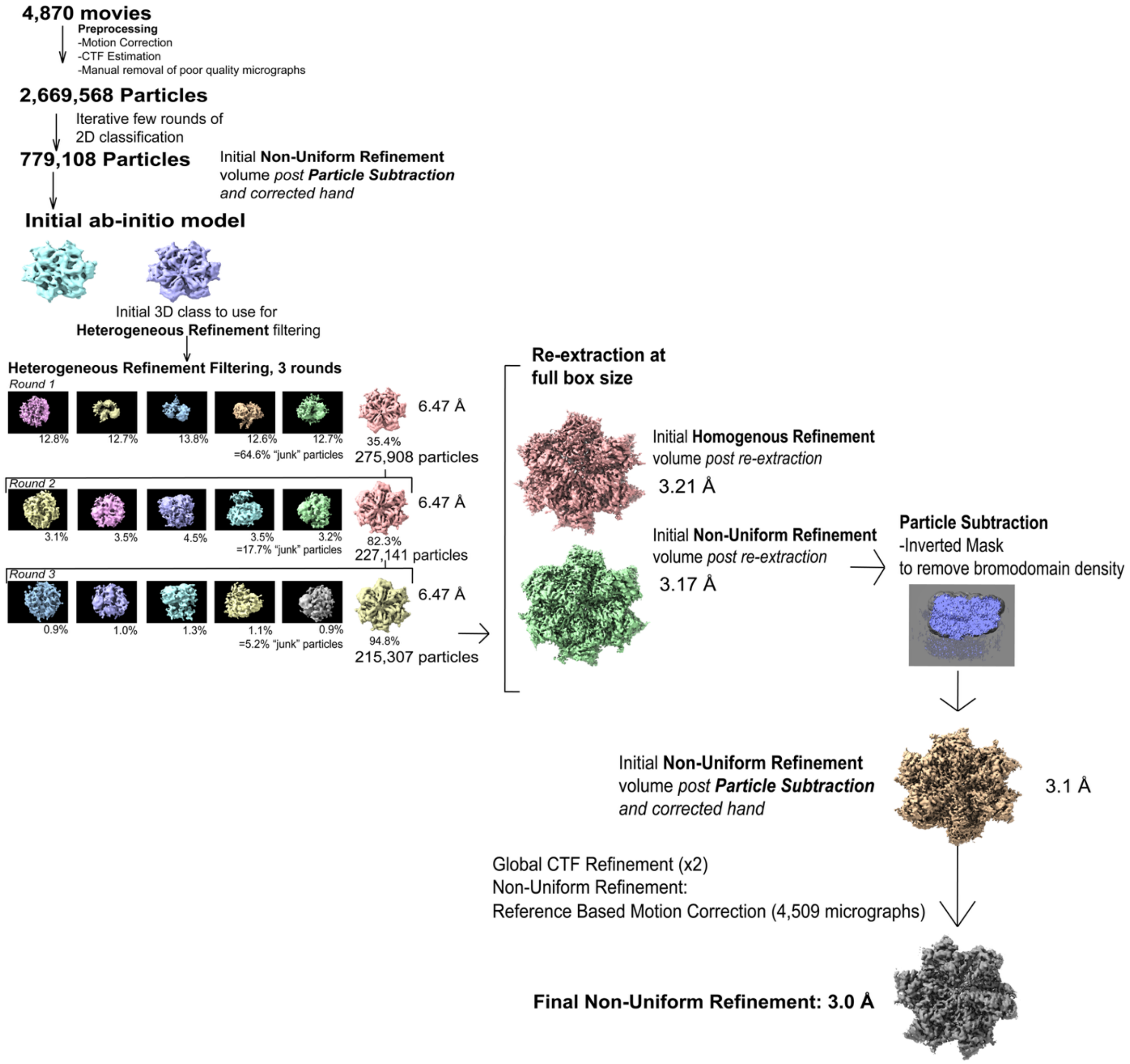

Supplementary Figure 3. Overview of the cryo-EM data processing workflow.
The single-particle cryo-EM data processing pipeline used to resolve structural heterogeneity and obtain a high-resolution reconstruction for the ATAD2B Walker B (E506Q) AAA+ ATPase bound to ATPγS and H4K5acK12ac, res 1-24. Following data collection, 4,870 micrographs were preprocessed using patch motion correction, CTF estimation, and particle picking. The resulting 2,669,568 particles were subjected to iterative rounds of two-dimensional (2D) classification, yielding 779,108 particles for downstream analysis. An initial *ab-initio* 3D reconstruction revealed substantial conformational and compositional heterogeneity. Particles were further sorted by multiple rounds of 3D heterogeneous refinement to filter out junk particles. The most homogenous and well resolved class was selected for re-extraction of the particles at the full box size, followed by homogenous refinement, non-uniform refinement, and particle subtraction. Global CTF refinement, non-uniform refinement and reference based motion correction were used to obtain the final 3D reconstruction of ATAD2B with an overall resolution of 3.0 Å. All data processing was carried out with cryoSPARC v.4.5.3[67].

bioRxiv preprint doi: https://doi.org/10.64898/2026.04.02.716110; this version posted April 3, 2026. The copyright holder for this preprint (which was not certified by peer review) is the author/funder, who has granted bioRxiv a license to display the preprint in perpetuity. It is made available under aCC-BY 4.0 International license.

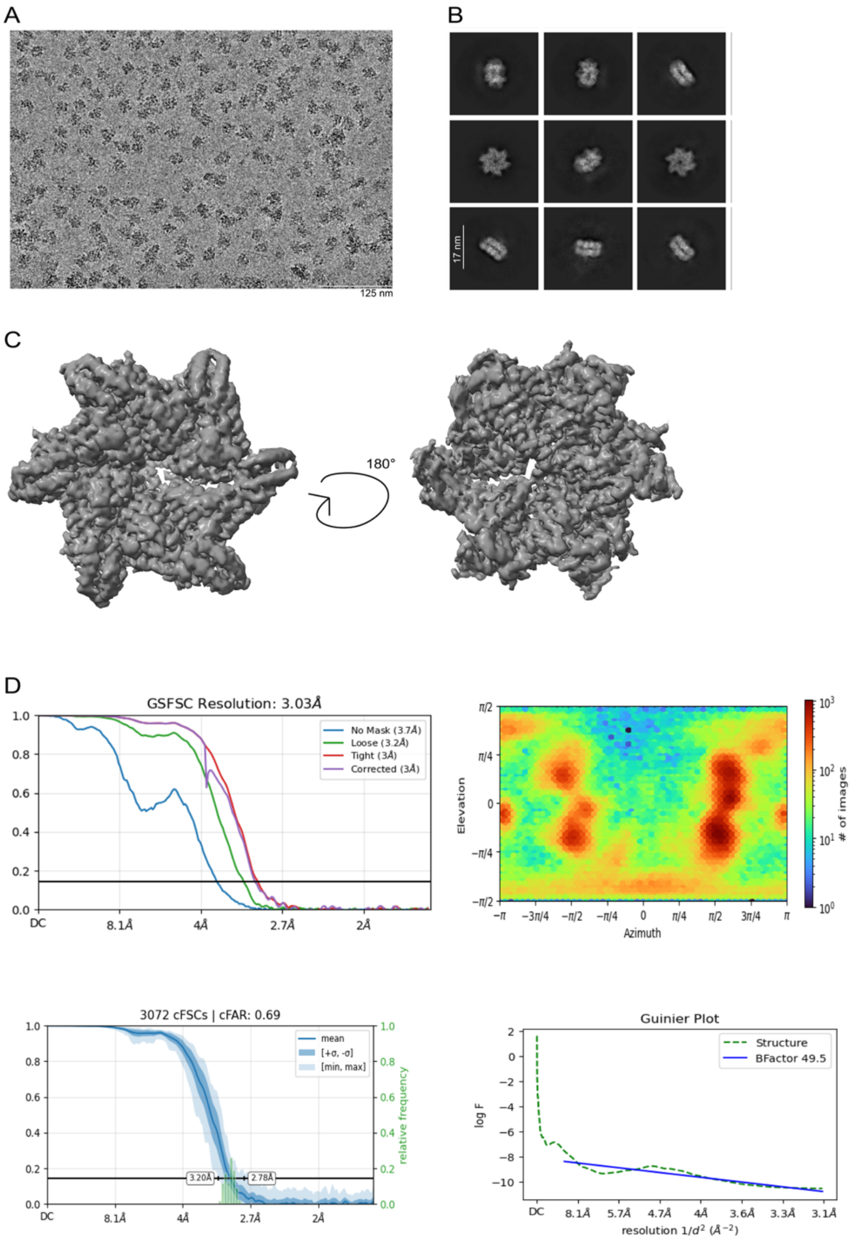

Supplementary Figure 4. Cryo-EM analysis and resolution assessment of the final ATAD2B Walker B reconstruction. a) Representative cryo-electron micrograph showing vitrified ATAD2B Walker B particles embedded in thin ice. Scale bar 125 nm. **b)** Representative 2D class averages from the final particle stack illustrating well-defined particle features in multiple orientations. Scale bar 17 nm. **c)** Final cryo-EM reconstruction of ATAD2B Walker B displaying top and bottom views of the hexameric complex. d) Gold-Standard Fourier Shell Correlation (GS-FSC) curves for the masked, unmasked, and corrected maps of ATAD2B Walker B, with an overall resolution of 3.0 Å at the FSC = 0.143 criterion. **e)** Euler angle distribution plot. **f)** The conical FSC (cFSC) plot reflecting the directional resolution anisotropy. The cFAR value of 0.69 indicates sufficient Fourier sampling. g) Guinier plot comparing the experimental structure factor with and without B-factor sharpening.

bioRxiv preprint doi: https://doi.org/10.64898/2026.04.02.716110; this version posted April 3, 2026. The copyright holder for this preprint (which was not certified by peer review) is the author/funder, who has granted bioRxiv a license to display the preprint in perpetuity. It is made available under aCC-BY 4.0 International license.

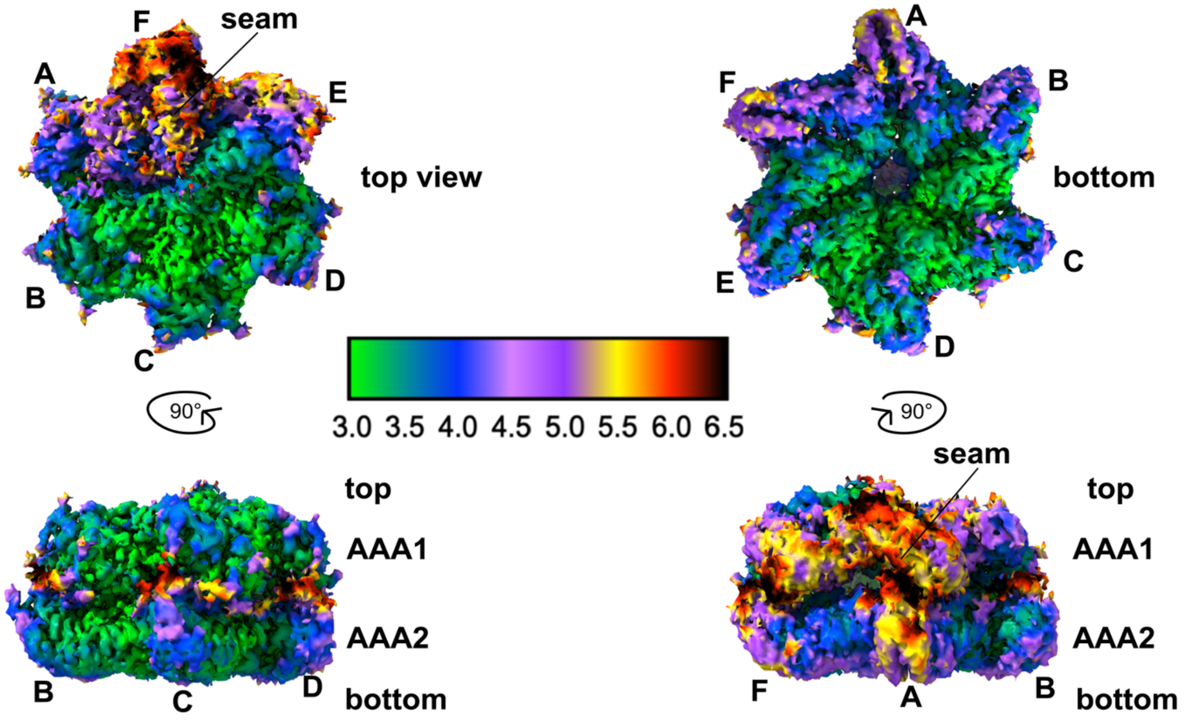

Supplementary Figure 5. Local resolution assessment of the ATAD2B cryo-EM reconstruction.
Top, bottom, interlocking side, bottom, and "seam" side views of the ATAD2B Walker B cryo-EM density map is colored by local resolution in ChimeraX. The local resolution estimation was performed in cryoSPARC v.4.5.3[67].

bioRxiv preprint doi: https://doi.org/10.64898/2026.04.02.716110; this version posted April 3, 2026. The copyright holder for this preprint (which was not certified by peer review) is the author/funder, who has granted bioRxiv a license to display the preprint in perpetuity. It is made available under aCC-BY 4.0 International license.

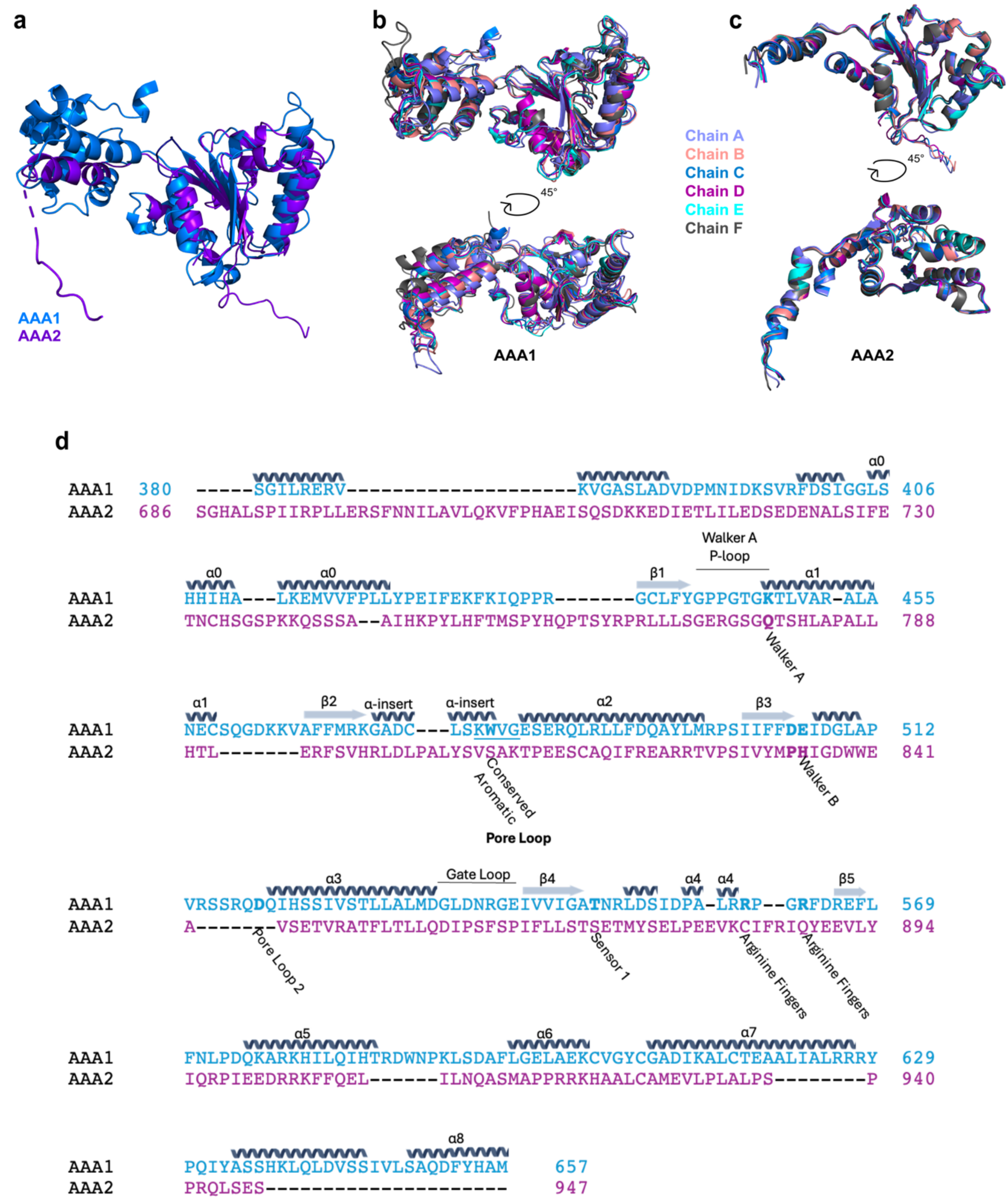

Supplementary Figure 6. Sequence alignment between the AAA1 and AAA2 domains of ATAD2B.
a) The AAA2 (purple) and AAA1 (blue) domains from subunit C of the ATAD2B Walker B structure are represented in cartoon and superimposed with each other. **b)** Each of the six AAA1 domains from the ATAD2B Walker B structure are superimposed with each other. Each subunit is colored as indicated. **c)** Each of the six AAA2 domains from the ATAD2B Walker B structure are superimposed with each other. Each subunit is colored as indicated. **d)** Multiple sequence alignment between the AAA1 (blue) and AAA2 (purple) domains of ATAD2B (UniProt ID: Q9ULI0). The domains (numbered as indicated) were aligned using the Clustal Omega Multi Sequence Alignment online tool (https://www.ebi.ac.uk/jdispatcher/msa/clustalo). Secondary structure prediction was performed using PROMALS3D[73]. The secondary structure elements are indicated above the sequence, and the conserved residues or structural motifs are labeled.

bioRxiv preprint doi: https://doi.org/10.64898/2026.04.02.716110; this version posted April 3, 2026. The copyright holder for this preprint (which was not certified by peer review) is the author/funder, who has granted bioRxiv a license to display the preprint in perpetuity. It is made available under aCC-BY 4.0 International license.

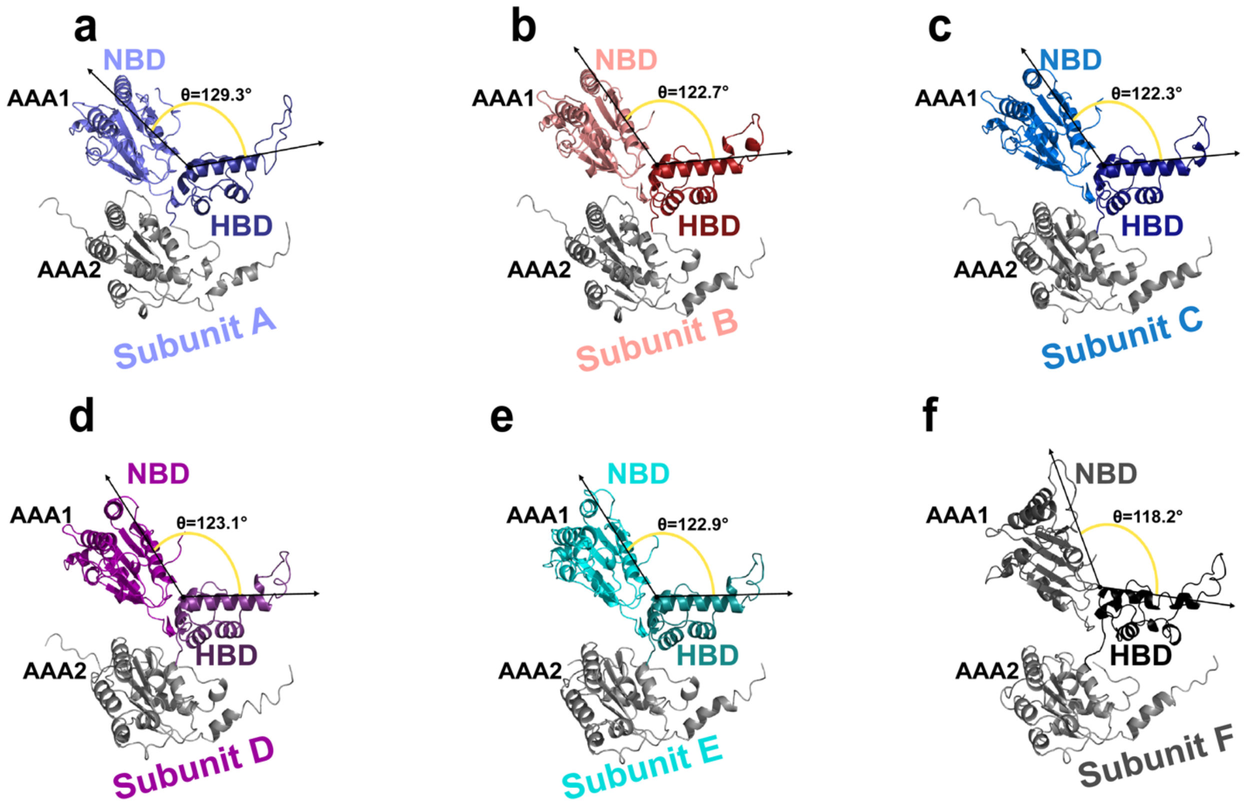

Supplementary Figure 7. Comparison of each ATAD2B Walker B subunit in the hexameric complex.
a-f) Each subunit is displayed in cartoon with the structural features labeled. The AAA1 domains are colored by subunit with subunit A: orchid, subunit B: salmon, subunit C: aqua, subunit D: plum, subunit E: turquoise, and subunit F: iron. In each AAA1 domain the nucleotide binding domain (NBD) is depicted in a lighter shade to the left, and the alpha-helical bundle domain (HBD) is depicted in a slightly darker shade of the same color on the right. All AAA2 domains are colored gray. The orientation of the NBD to the HBD in each AAA1 domain of ATAD2B Walker B was measured using residues 625-611-479, and is represented by two black arrows connected by a yellow curve. The measurement of the angle is displayed next to it and marked with a θ.

bioRxiv preprint doi: https://doi.org/10.64898/2026.04.02.716110; this version posted April 3, 2026. The copyright holder for this preprint (which was not certified by peer review) is the author/funder, who has granted bioRxiv a license to display the preprint in perpetuity. It is made available under aCC-BY 4.0 International license.

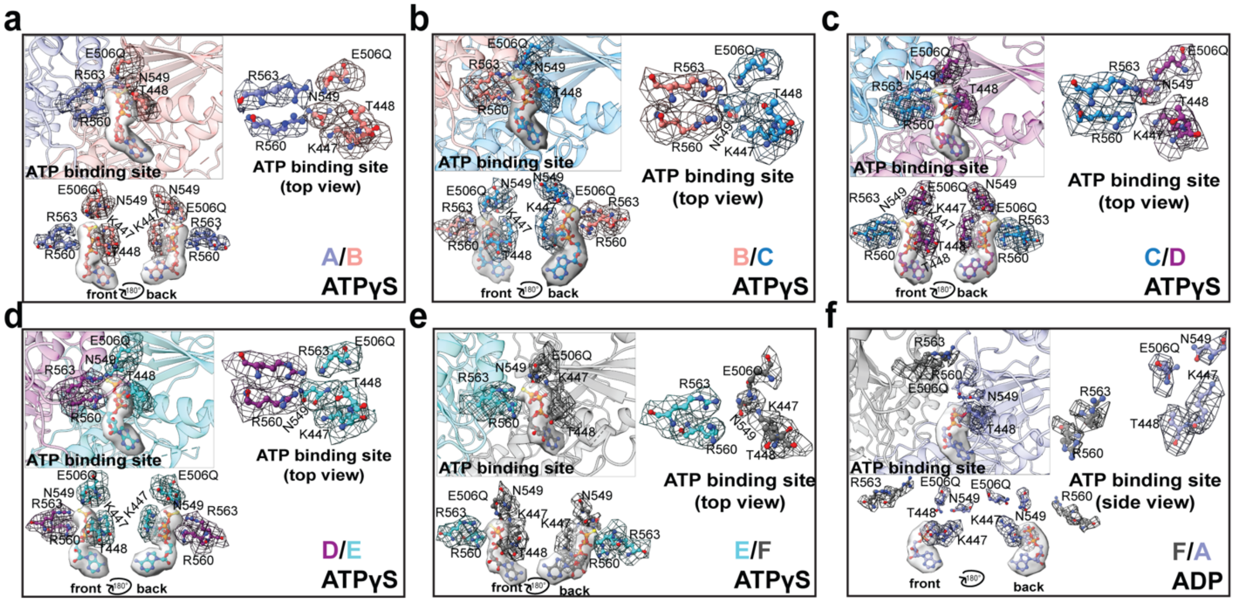

Supplementary Figure 8. Density in the nucleotide binding site surrounding ATPγS or ADP.
a-f) Conserved AAA+ ATPase residues as shown as sticks and include Walker A (K447), T448, Walker B (E506Q), Sensor 1 (N549), and the arginine finger residues (R560 and R563). The cryo-EM density map was contoured at 0.0202 using ChimeraX. For each nucleotide, the density is shown as surface (gray, 41% transparent). For each conserved residue, the density is displayed as mesh and colored the same as its representative subunit according to the following scheme: subunit A: orchid, subunit B: salmon, subunit C: aqua, subunit D: plum, subunit E: turquoise, subunit F: iron. In each panel (a-f), the top left image is a view of the entire ATP binding pocket with the respective subunits shown in cartoon and the conserved residues are shown as sticks. In each panel (a-f), the top right image is a view of the conserved amino acid residues involved in nucleotide coordination without the nucleotide present for better visualization of the density (mesh). In each panel (a-f), the bottom left image is a view of the ATPγS or ADP nucleotide and the conserved amino acid residues involved in nucleotide coordination. Two views are shown, one from the front of the binding site, and another rotated by 180° to show the back for full visualization of each conserved residue.

bioRxiv preprint doi: https://doi.org/10.64898/2026.04.02.716110; this version posted April 3, 2026. The copyright holder for this preprint (which was not certified by peer review) is the author/funder, who has granted bioRxiv a license to display the preprint in perpetuity. It is made available under aCC-BY 4.0 International license.

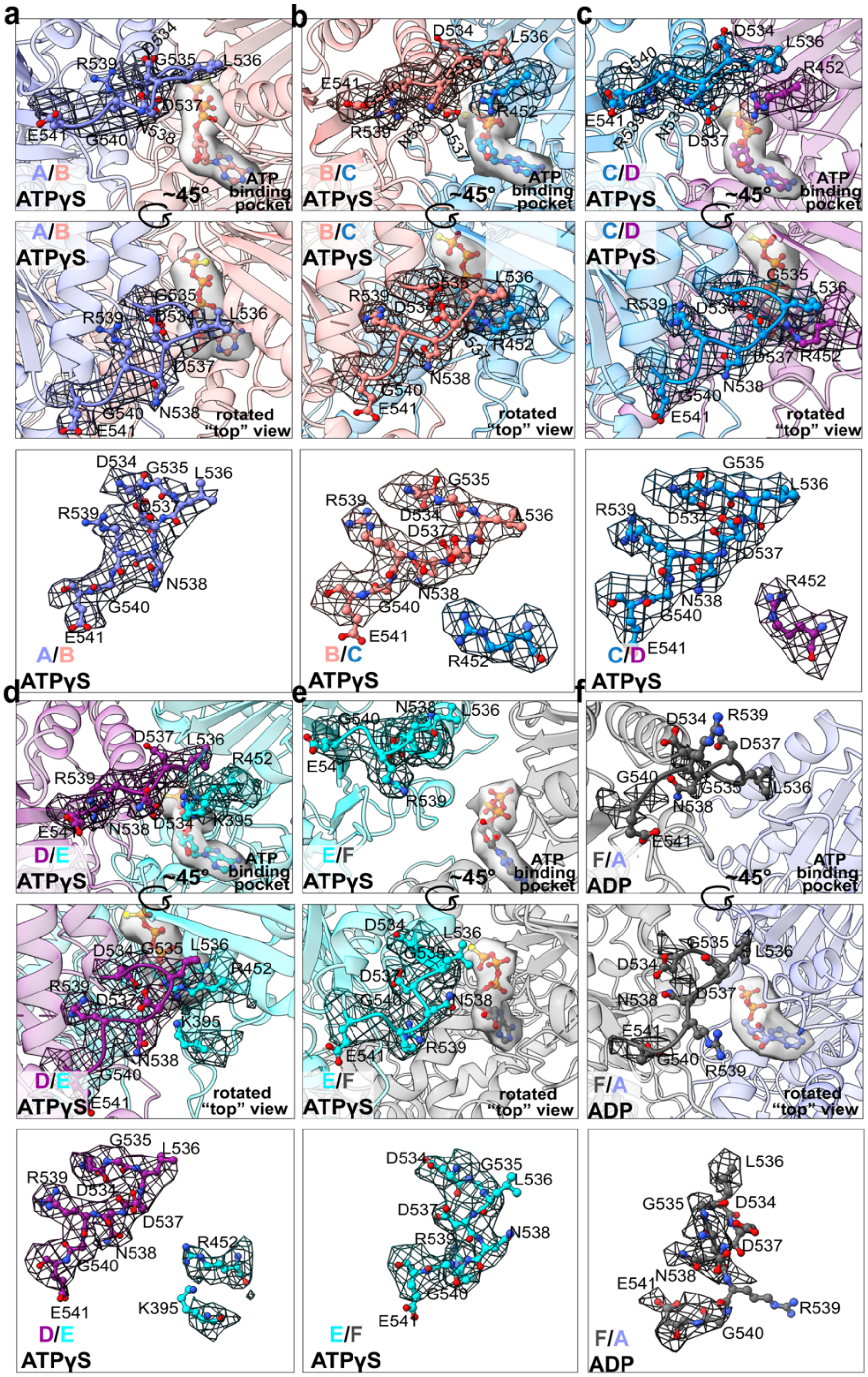

Supplementary Figure 9. Asymmetric gate loop conformations in the ATAD2B Walker B hexamer.
(a-f) Gate loop residues (D534-E541) and interacting residues (R452 and K395) when applicable are shown as sticks. The density map was contoured at 0.0202 in ChimeraX. For each nucleotide, the density is shown as surface (gray, 41% transparent). For each conserved residue, the density is shown as mesh and colored the same as its representative subunit. Subunit A: orchid, subunit B: salmon, subunit C: aqua, subunit D: plum, subunit E: turquoise, subunit F: iron. Three views are shown for a-f, where the top panel is a side view of the gate loop looking into the nucleotide binding pocket. The middle panel is the same gate loop rotated by ~45° to look into the nucleotide binding pocket. Subunits A-D have "closed" gate loops, and subunits E-F have "open" gate loops. The bottom panel shows the density surrounding the gate loop residues and any nucleotide binding residues when applicable for better visualization of the density.

bioRxiv preprint doi: https://doi.org/10.64898/2026.04.02.716110; this version posted April 3, 2026. The copyright holder for this preprint (which was not certified by peer review) is the author/funder, who has granted bioRxiv a license to display the preprint in perpetuity. It is made available under aCC-BY 4.0 International license.

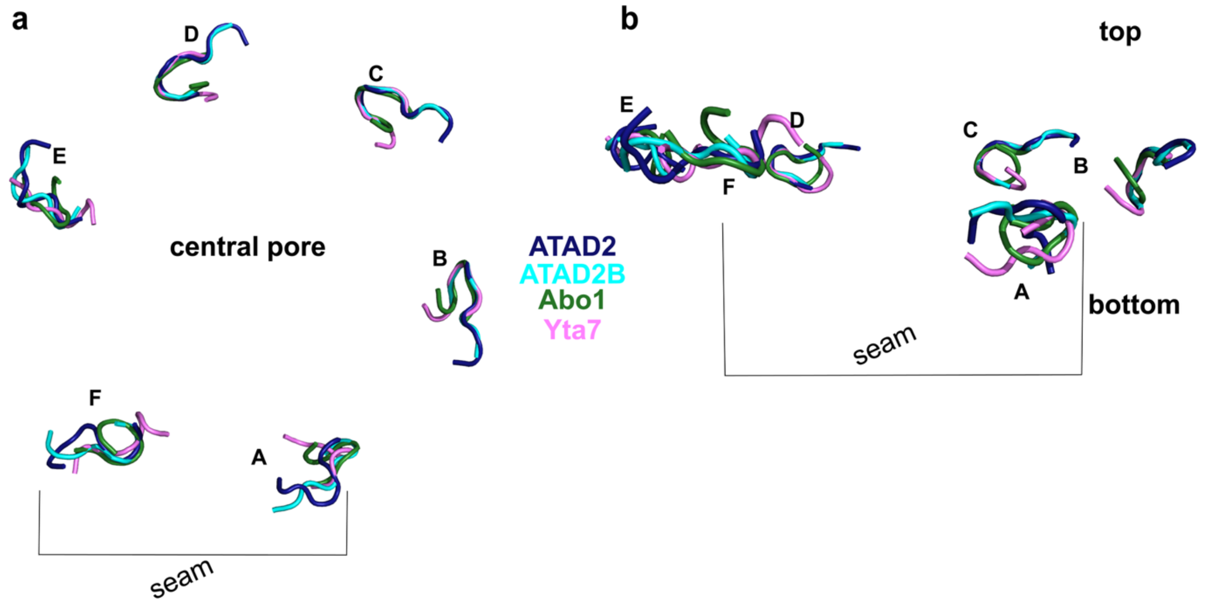

Supplementary Figure 10. Comparative analysis of the AAA1 domain gate loops across the ATAD2-lke family. a-b) ATAD2B (residues 534-541) was aligned with homologs with solved structures at the gate loops: ATAD2 (PDB ID: 8h3h)(residues 560-568), Abo1 (PDB ID: 6jpu)(residues 398-405), Yta7 (PDB ID: 7uqj)(residues 545-552). These superimpositions are represented as cartoons and colored as indicated: ATAD2B: by subunit, ATAD2: density, Abo1: forest green, Yta7: pink. **a)** Top view of the aligned gate loops from the AAA1 domain. **b)** Side view of the aligned gate loops from the AAA1 domain. Offset between the seam protomers are labeled.

bioRxiv preprint doi: https://doi.org/10.64898/2026.04.02.716110; this version posted April 3, 2026. The copyright holder for this preprint (which was not certified by peer review) is the author/funder, who has granted bioRxiv a license to display the preprint in perpetuity. It is made available under aCC-BY 4.0 International license.

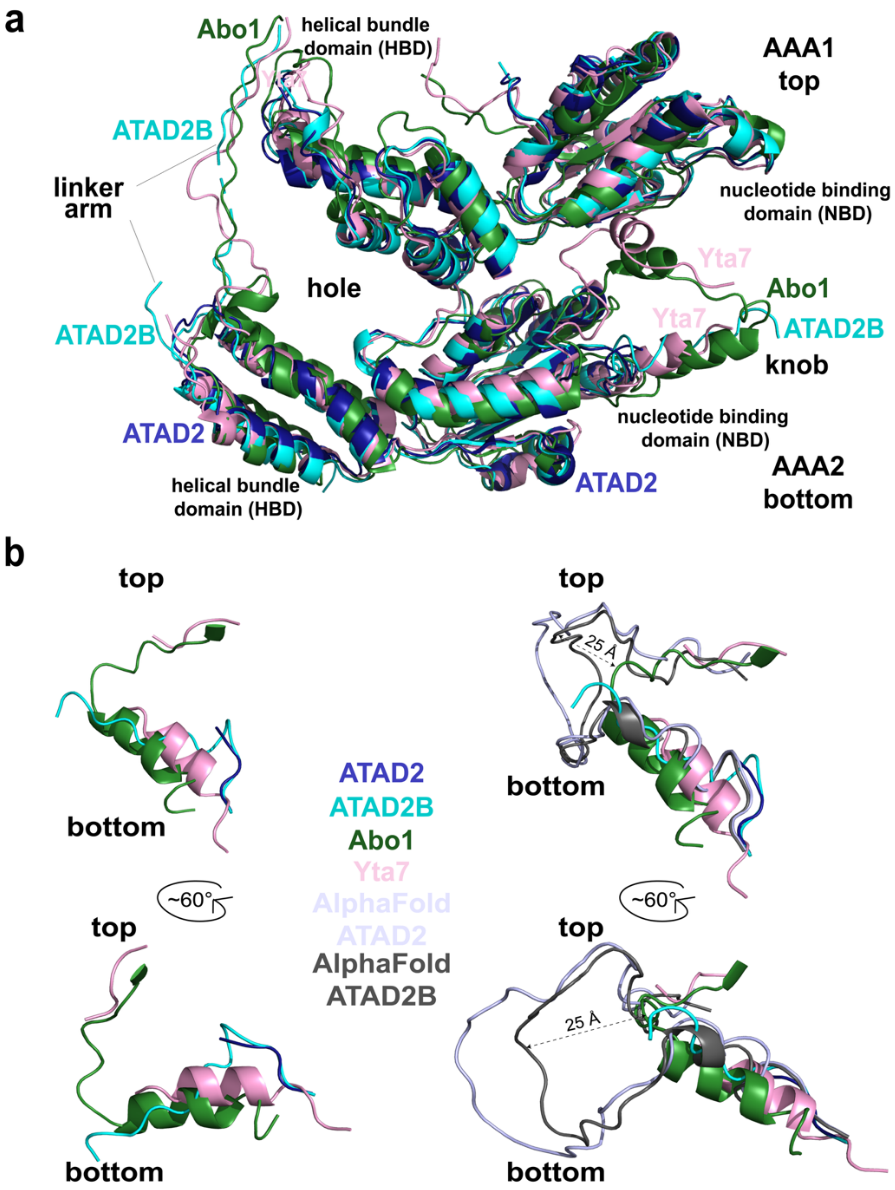

Supplementary Figure 11. Structural comparison of the knob-hole and linker arm interactions between the ATAD2-like family AAA+ ATPases. a-d) The most well-resolved subunits from the ATAD2B Walker B structure (subunits C and D) were structurally aligned to the corresponding subunits in ATAD2 (PDB ID: 8h3h), Abo1 (PDB ID: 6jpu), Yta7 (PDB ID: 7uqj) using PyMOL[74]. **a)** A surface view of each structure is shown with ATAD2 in purple, ATAD2B in marine, Abo1 in dark blue, and Yta7 in light blue. The knob region from the C subunit of each protein is displayed in cartoon. **b)** The hole is formed by the stacked helical bundle domains of AAA1 and AAA2 along with linker arm from the adjacent subunit (counter clockwise). A cartoon representation of the superimposed D subunits is shown with ATAD2 in hot pink, ATAD2B in purple, Abo1 in violet purple, and Yta7 in light pink. The knob region is also labeled. **c)** The knob-hole region of the ATAD2-like family proteins is depicted. The C and D subunits are shown in surface view with only the knob residues shown in cartoon. The C subunits are colored for each protein with ATAD2 in purple, ATAD2B in marine, Abo1 in blue, and Yta7 in light blue. The knobs extend into the hole formed by subunit D in each protein, which is colored with ATAD2 in hot pink, ATAD2B in purple, Abo1 in violet purple, and Yta7 in light pink. **d)** The left side depicts the knob region of experimentally determined structures. It shows a structural alignment of the knob region from subunit C of each protein with ATAD2 in dark blue, ATAD2B in marine, Abo1 in purple, and Yta7 in green. The right side is the same, but also includes AlphaFold models of ATAD2B (AF-Q9ULI0-F1-v4) in orange, and ATAD2 (AF-Q6PL18-F1-v4) in cyan[44].

bioRxiv preprint doi: https://doi.org/10.64898/2026.04.02.716110; this version posted April 3, 2026. The copyright holder for this preprint (which was not certified by peer review) is the author/funder, who has granted bioRxiv a license to display the preprint in perpetuity. It is made available under aCC-BY 4.0 International license.

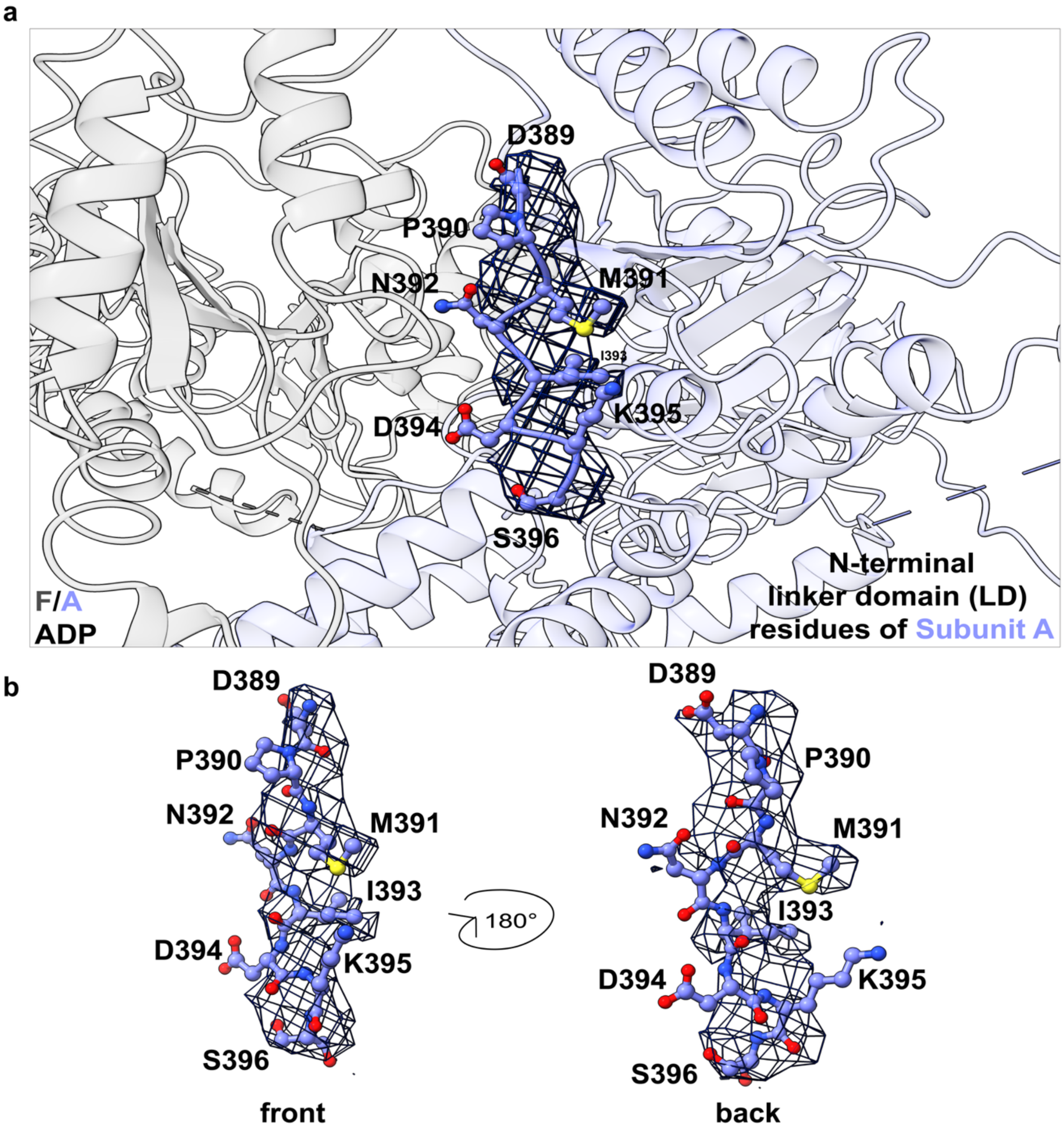

Supplementary Figure 12. Cryo-EM density of the ATAD2B Walker B linker domain.
a) The cryo-EM density map of the ATAD2B Walker B-ATPγS-H4K5acK8ac structure was contoured at 0.0202 in ChimeraX. Subunits F (iron) and A (orchid) are represented in cartoon, with residues 389-396 from the linker domain of subunit A shown as sticks. The density map surrounding the linker domain residues is shown as a black mesh around each residue. **b)** Stick representation of the linker domain residues from subunit A with two views rotated by 180° for better density visualization.

bioRxiv preprint doi: https://doi.org/10.64898/2026.04.02.716110; this version posted April 3, 2026. The copyright holder for this preprint (which was not certified by peer review) is the author/funder, who has granted bioRxiv a license to display the preprint in perpetuity. It is made available under aCC-BY 4.0 International license.

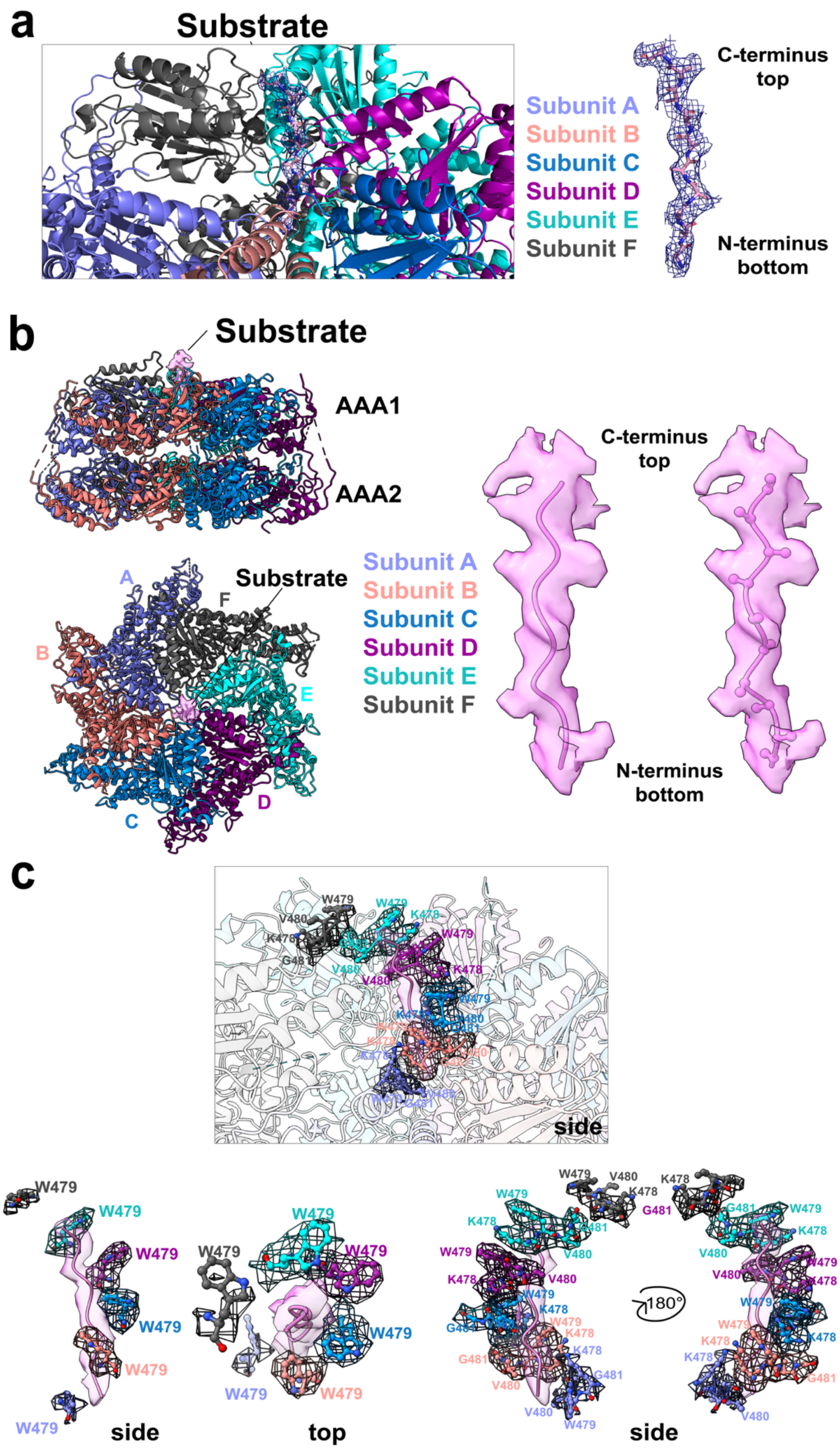

bioRxiv preprint doi: https://doi.org/10.64898/2026.04.02.716110; this version posted April 3, 2026. The copyright holder for this preprint (which was not certified by peer review) is the author/funder, who has granted bioRxiv a license to display the preprint in perpetuity. It is made available under aCC-BY 4.0 International license.

Supplementary Figure 13. Cryo-EM density of substrate and pore loop residues in ATAD2B Walker B. **a-c)** Density for subunits A-E was contoured to a level of 0.0202 in ChimeraX, while the density for the more flexible seam subunit F was contoured to a level of 0.0149 for clarity. **a)** Left panel, angled view of the ATAD2B Walker B structure (in cartoon) highlighting density in the pore with each subunit colored as indicated. Right panel, cryo-EM density for the substrate observed in the central pore is shown in blue mesh, and the poly-alanine residues of the modeled substrate are shown in sticks. **b)** Left panel, top and side views of the ATAD2B Walker B structure shown in cartoon. Right panel, left and right images of the cryo-EM density in the central pore shown in pink with cartoon and stick representations of the poly-alanine chain built in the pore. **c)** Top center panel, side view of the tryptophan staircase surrounding the substrate in the central pore region of ATAD2B. The conserved tryptophan residue (W479 in AAA1) is shown in sticks for each subunit in the ATAD2B hexamer and the cryo-EM density surrounding each residue is shown as a black mesh. Bottom panels, show side and top views of the substrate in the pore (cartoon) built into the cryo-EM density (pink). The side view on the right includes the four conserved pore loop residues KWVG (479-481) that contact the substrate with the density in black mesh. The two views of the pore loop residues surrounding the substrate are rotated by 180° for better density visualization.
